# Non‐invasive ventilation and airway exchange catheter using a novel adapter in a difficult airway patient with post‐extubation respiratory failure

**DOI:** 10.1002/rcr2.558

**Published:** 2020-04-18

**Authors:** Oscar Ivan Quintero Osorio, Janer V. Arenas, Juan A. Cuervo, Gustavo A. Ospina Tascón

**Affiliations:** ^1^ Fundación Valle del Lili Centro de Investigaciones Clínicas Cali Colombia; ^2^ Department of Intensive Care Medicine Fundación Valle del Lili Cali Colombia; ^3^ Department of Anesthesiology Fundación Valle del Lili Cali Colombia; ^4^ Facultad de Ciencias de la Salud Universidad Icesi Cali Colombia

**Keywords:** Airway exchange catheter, difficult airway, nasoenteric tubes, non‐invasive ventilation, post‐extubation respiratory failure

## Abstract

Post‐extubation respiratory failure in patients with difficult airway is considered a challenge for the health team. Some intratracheal devices such as airway exchange catheters (AECs) could be used during scheduled tube removing to ensure a rapid access to airway in the case of requiring emergent reintubation. Nevertheless, using such devices could impede adequate non‐invasive mechanical ventilation (NIMV) support because of the air leaks generated by interfering with mask interfaces. We describe the case of a woman with a very difficult airway in whom an AEC was placed before scheduled extubation and then developed post‐extubation respiratory failure. Mask interface was adequately sealed by using a novel tube adapter for NIMV and successful non‐invasive ventilation was provided while maintaining the AEC placed in the trachea until the emergency reintubation risk was overcome.

## Graphical abstract

We describe the case of a woman with a difficult airway who developed post‐extubation respiratory failure. We provided successful non‐invasive ventilation support using a novel tube adapter for non‐invasive mechanical ventilation, which improved the patient's adaptation to a nasoenteric tube and an oronasal mask. Simultaneously, an airway exchanger catheter placed in the trachea was maintained in situ until emergency reintubation risk was overcome.

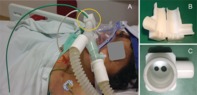

## Introduction

Extubation of patients with a difficult airway in the intensive care unit (ICU) or anaesthesia setting represents a challenge for the health team considering the consequences of a potential loss of control of the airway. Use of airway exchange catheters (AECs) is currently recommended during scheduled extubation in patients combining a very difficult airway and high risk of reintubation 1. Although the use of non‐invasive mechanical ventilation (NIMV) in post‐extubation respiratory failure is very common, its combined use with AECs is not. Even though placing an AEC device might increase possibilities to recover the control of airway in the case of needing reintubation, AEC itself could lead to the failure of NIMV due to air leaks induced by an inadequate sealing of NIMV mask interfaces. We present an example of successful NIMV support in a case of post‐extubation respiratory failure whilst an AEC was simultaneously maintained in the trachea, using a new tube adapter for the NIMV (TA‐NIMV) interface.

## Case Report

**Figure 1:**
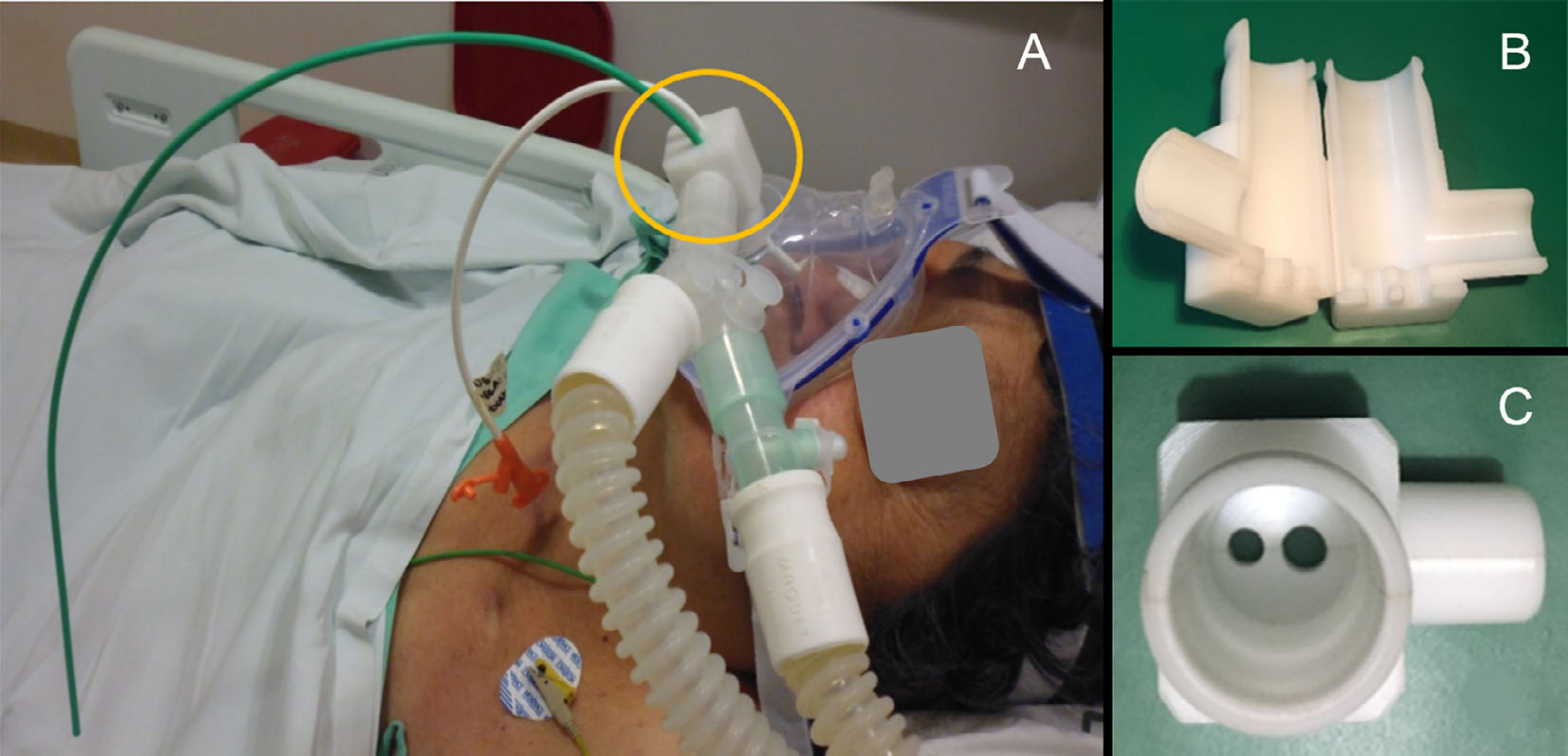
(A) Non‐invasive mechanical ventilation (NIMV) support using a double‐lumen tube adapter for NIMV (TA‐NIMV) to fix oronasal mask to the ventilator with simultaneous use of airway exchange catheter (AEC) and nasoenteric tube (yellow circle). (B) Open TA‐NIMV. (C) Closed TA‐NIMV.

A 64‐year‐old woman was admitted to the emergency room because of the complaints of chills and fever. She developed a rapidly progressive and severe inflammatory process in the left inguinal region. She had a history of hypertension, hypothyroidism, and grade III obesity (body mass index >40 kg/m^2^). In addition, she was a chronic steroid user because of an antecedent of rheumatoid arthritis. Upon admission, the patient developed hypotension despite receiving adequate fluid resuscitation and finally required vasoactive support. A necrotizing fasciitis was diagnosed. Wide‐spectrum antibiotic therapy was started and she was transferred to the operating room for surgical exploration and source control. A difficult airway was predicted because of Mallampati Class III, Cormack–Lehan Class III upon laryngoscopy, thyromental distance Class III, and mandibular protrusion Class III. She was intubated with some difficulty under endoscopic assistance. An extensive surgical debridement was performed and a vacuum‐assisted closure system was placed. Thereafter, she was transferred to the ICU where goal‐directed resuscitation, mechanical ventilation, and general support were continued. Haemodynamic and ventilatory parameters considerably improved after six days of medical treatment and additional surgical interventions. At such point, the ICU team started the weaning of mechanical ventilation. After fulfilling all criteria, extubation was scheduled. Being aware of the very difficult airway, an AEC (double‐lumen, soft‐tipped extra firm catheter, 12‐French, 2.3‐mm internal diameter, 100‐cm length; Cook® Medical, USA) was placed in the trachea, and the endotracheal tube was removed. Oxygen was provided using a venturi mask at an inspired oxygen fraction of 0.50, while light sedation was provided with dexmedetomidine. After 30 min, the patient exhibited signs of respiratory failure. Because of the presence of both AEC and nutrition enteral tubes, adaptation to the NIMV mask was not adequate. A significant air leak was detected despite the leak compensation provided by the ventilator (Servo‐i® ventilator; Maquet Critical Care, Sweden; NIMV pressure control mode). Therefore, a double‐lumen TA‐NIMV device was placed at the end of the interface mask (Acucare™ F1‐0 Hospital Non‐Vented Full Face Mask; ResMed Ltd, USA) (Fig. 1A), while NIMV was provided using pressure control mode: pressure control: 7 cm H_2_O; positive end‐expiratory pressure (PEEP): 5 cm H_2_O; expiratory volume: 359 mL; peak inspiratory pressure: 15 cm H_2_O; inspiratory time: 1.00; inspiratory–expiratory (I:E) ratio of 1:3; respiratory ratio: 20 rpm; and fraction of inspired oxygen (FiO_2_): 0.40. After placing the TA‐NIMV, the percentage of air leakage was always <10%. The AEC was maintained in situ during the next 6 h, when the respiratory situation was considered to be resolved. After this period, the AEC device was removed from the trachea and during the subsequent 24 h, intermittent NIMV was provided using a single‐lumen TA‐NIMV as the enteral nutrition tube was maintained. She did not require reintubation, and she was finally discharged from the ICU in good condition.

## Discussion

We report the case of post‐extubation respiratory failure in a patient with a difficult airway who received successful NIMV support while an AEC was simultaneously maintained in the trachea by using a new adapter between the facial mask interface and the ventilator.

Up to 20% of patients receiving ventilation support in the ICU will require reintubation despite achieving an adequate spontaneous breathing trial 2. In this scenario, the use of NIMV has shown to have a role in the post‐extubation respiratory failure preventing reintubation in some special populations, and in cases of post‐extubation hypercapnic respiratory failure 3. Nevertheless, despite the benefits of using NIMV during hypoxaemic failure, its role during hypoxaemic post‐extubation respiratory failure remains less evident. Use of AEC has been related with increased risk of tracheal perforation, interstitial pulmonary emphysema, and dislodgement 4. However, its use is still recommended in the current guidelines of tracheal intubation 1.

Using the TA‐NIMV allowed to provide adequate NIMV support by significant reduction of the percentage of air leak not compensated by the ventilator non‐invasive mode. Such TA‐NIMV device might improve comfort and reduce air leaks during NIMV support 5. Briefly, this device is a simple piece assembled in two halves (Fig. 1B) with one or two holes at the bottom (Fig. 1C) that facilitates the passage of tubes of different sizes.

The use of interface adapters as the TA‐NIMV could decrease air leaks during NIMV, thus improving the respiratory mechanics. Nevertheless, the structure of the TA‐NIMV could be potentially disassembled during the period in which NIMV is being provided, which should force to keep a close monitoring during its use. The use of interface adapters as the TA‐NIMV during post‐extubation respiratory failure should be evaluated in the future.

### Disclosure Statement

Appropriate written informed consent was obtained for publication of this case report and accompanying images.

Ethical approval was obtained from the institution Ethics Committee, protocol number 00708/16. Fundación Valle del Lili has the device patent of the TA‐NIMV. The patent number 1165 is registered at the Colombian Patent and Trademark Office (Superintendencia de Industria y Comercio de la República de Colombia). International publication is WO2014188242A1 of World Intellectual Property Organization.
